# Inflammatory segmental vitiligo during oral isotretinoin use: a casual association?^☆^^☆☆^

**DOI:** 10.1016/j.abd.2019.07.014

**Published:** 2020-03-20

**Authors:** Maria Fernanda de Santana Avelar-Caggiano, Caio César Silva de Castro, Gerson Dellatorre

**Affiliations:** a Service of Dermatology, Hospital Santa Casa de Misericórdia de Curitiba, Curitiba, PR, Brazil; b Medical School, Pontifícia Universidade Católica do Paraná, Curitiba, PR, Brazil

*Dear Editor*,

Segmental vitiligo (SV) accounts for 3–20% of all cases of vitiligo and is usually not associated with autoimmune diseases when compared to non-segmental vitiligo (NSV).^1^ Among the theories proposed for the path physiology of SV, it is essential to emphasize the presence of an autoimmune attack against a mosaic area.^1^

In the literature, there are few reports on the emergence of vitiligo as a side effect of medications, especially oral isotretinoin. The objective of the present report is to demonstrate a possible new side effect of this medication since there are no descriptions of its association with SV in the literature.

A 17 year-old male patient, previously healthy and without a family history of vitiligo, was diagnosed with resistant acne, previously treated with topical and systemic antibiotic therapy. During the fifth month of treatment with oral isotretinoin (0.4 mg/kg/day, cumulative dose of 5.400 mg), there were a chromic spots surrounded by an erythematous-scaling halo in malar and perioral areas, not exceeding the mid line on the right side of the face (Fig. 1). Wood's light examination revealed fluorescent chalky white aspect, as well as polios is in beard hair (Fig. 2) favoring the SV diagnosis. After the withdrawal of isotretinoin, the patient began treatment with 0.1% tacrolimus ointment twice daily with an improvement of perilesional erythema after two months, although without improvement of achromy. Subsequently, it was submitted to 20 sessions of UVB-NB phototherapy, with little perifollicularre pigmentation.

**Figure 1:**
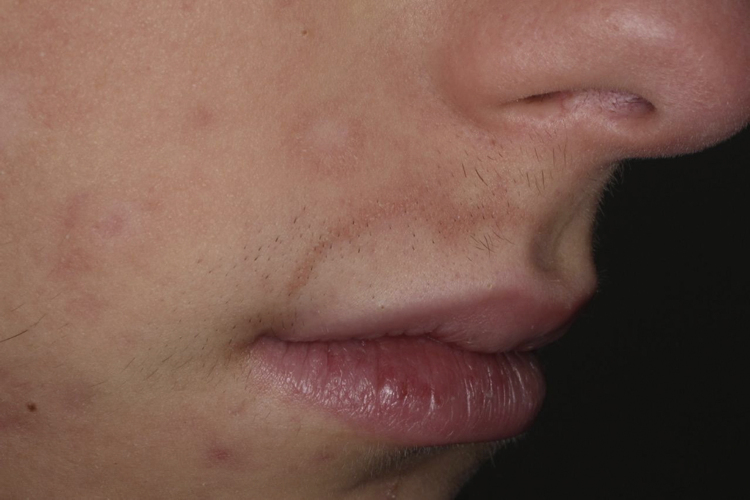
Achromic spots surrounded by halo erythematosus and polios is in beard hairs in the malar and right perioral regions.

**Figure 2:**
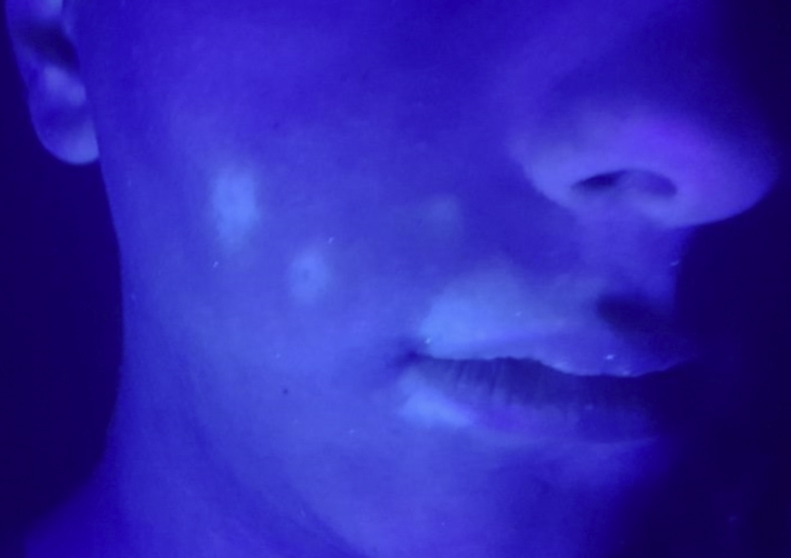
Wood's light examination revealed fluoresce bright blue-white in malar and perioral right regions.

In the literature review, only three cases of vitiligo in the context of oral isotretinoin use are described. One report describes the case of a patient who developed vitiligo during the use of oral isotretinoin at a dose of 0.3–0.4 mg/kg/day for the treatment of moderate to severe acne.^2^ In another report, acrofacial vitiligo was developed only two months after the end of treatment with isotretinoin, which reduces the probability of a cause-and-effect relationship.^3^ There is also a report of worsening of NSV lesions in the lower lip and lower perioral region after chronic cheilitis due to the use of oral isotretinoin; a condition attributed in this case to the Koebner phenomenon.^4^

The mechanism of action of isotretinoin in this presumed association is not yet fully elucidated, but the drug appears to play a role in triggering autoimmunity in genetically susceptible individuals.^5^ Several reports of autoimmune diseases such as diabetes, autoimmune hepatitis, Guillain–Barré syndrome, and thyroiditis have been reported after the end of the isotretinoin regimen or during the last week of treatment.^5^ Besides, in vitro studies have also demonstrated that retinoid may have a pro-apoptotic effect on melanocytes.^3^

Although the cause-effect relationship of this association has not yet been proven, the increasing appearance of new cases in the literature is a warning sign for dermatologists to keep vigil on this possible new side effect.

## Financial support

None declared.

## Authors' contributions

Maria Fernanda de Santana Avelar-Caggiano: Conception and planning of the study; elaboration and writing of the manuscript; obtaining, analysis, and interpretation of the data; critical review of the literature.

Caio César Silva de Castro: Approval of the final version of the manuscript; effective participation in research orientation; intellectual participation in the propaedeutic and/or therapeutic conduct of the studied cases; critical review of the manuscript.

Gerson Dellatorre: Approval of the final version of the manuscript; effective participation in research orientation; intellectual participation in the propaedeutic and/or therapeutic conduct of the studied cases; critical review of the manuscript.

## Conflicts of interest

None declared.
